# Supplementary material for: Target DNA bending by the Mu transpososome promotes careful transposition and prevents its reversal
Source: eLife. 2017 Feb 13;6:e21777. doi: 10.7554/eLife.21777 (PMC5357137; doi:10.7554/eLife.21777)
Supplement: Figure 6—source data 1. — TCSPC FRET data are organized into folders by date of collection so that the data can be paired with their proper instrument response function (IRF). For each measurement taken, three files are given: (1). ifx: The raw output data from the Vinci fluorometer control software (2). txt: A tab delimited text representation of the data, first column is time (ns) and the second is photon counts (3). pdf: The output of the lifetime fitting done in the Vinci control software and represented in Figure 6. Filenames are descriptive of the sample and conditions: WT or D3: Wild-type transpososomes, or SinMu transpososomes lacking domain III on the catalytic subunits. DMSO: Where present, indicates 15% (v/v) DMSO in the buffer. Mismatch: Where present, indicates that the labeled target DNA contained the central G:G base pairing mistmatch. DO or DA: Target DNA was singly labeled with donor Atto565 fluorophore, or labeled with both donor Atto565 and acceptor Atto647N at opposite ends. 60c: Where present, indicates that the sample and vessel were heated to 60 degrees celsius prior to measurement. DOI: http://dx.doi.org/10.7554/eLife.21777.018 [file elife-21777-fig6-data1.docx]

|  | | | | | **Gaussian fraction** | | | | | **Discrete fraction** | | |
| --- | --- | --- | --- | --- | --- | --- | --- | --- | --- | --- | --- | --- |
| **Fluorophore configuration** | **Sample** | **Target DNA mismatch** | **DMSO** | **χ^2^** | **Tau (ns)** | **± error** | **FWHM (ns)** | **± error** | **α** | **Tau (ns)** | **± error** | **α** |
| DO | DNA only | No | No | 1.41 | 1.66 | 0.1 | 1.09 | 0.3 | 2.04 | 4.68 | 0.007 | 20.6 |
| DA | DNA only | No | No | 1.35 | 1.51 | 0.1 | 0.666 | 0.4 | 2.25 | 4.54 | 0.006 | 21.2 |
| DO | DNA only | No | Yes | 1.42 | 1.74 | 0.2 | 1.23 | 0.3 | 2.19 | 4.5 | 0.007 | 21.3 |
| DA | DNA only | No | Yes | 1.37 | 1.73 | 0.2 | 1.17 | 0.3 | 2.37 | 4.36 | 0.007 | 21.9 |
| DO | DNA only | Yes | Yes | 1.44 | 1.30 | 0.1 | 0.87 | 0.3 | 1.59 | 4.45 | 0.005 | 22.0 |
| DA | DNA only | Yes | Yes | 1.35 | 1.73 | 0.2 | 1.50 | 0.3 | 1.96 | 4.34 | 0.007 | 22.3 |
| DO | DNA only, 60C | Yes | Yes | 1.29 | 1.66 | 0.2 | 0.674 | 0.7 | 1.86 | 4.34 | 0.008 | 22.3 |
| DA | DNA only, 60C | Yes | Yes | 1.22 | 2.23 | 0.4 | 2.1 | 0.5 | 2.38 | 4.26 | 0.01 | 22.2 |
| DO | WT TCC | No | No | 1.36 | 1.71 | 0.1 | 0.979 | 0.3 | 2.23 | 4.7 | 0.007 | 20.4 |
| DA | WT TCC | No | No | 1.35 | 3.31 | 0.2 | 2.06 | 0.2 | 11.2 | 4.54 | n/a | 13.5 |
| DO | WT TCC | No | Yes | 1.40 | 1.91 | 0.2 | 1.68 | 0.3 | 2.30 | 4.51 | 0.008 | 21.1 |
| DA | WT TCC | No | Yes | 1.50 | 3.30 | 0.3 | 1.72 | 0.3 | 16.8 | 4.36 | n/a | 9.76 |
| DO | WT TCC | Yes | Yes | 1.33 | 2.30 | 0.3 | 1.97 | 0.5 | 2.25 | 4.53 | 0.01 | 20.9 |
| DA | WT TCC | Yes | Yes | 1.58 | 3.34 | 0.2 | 1.57 | 0.2 | 24.2 | 4.33 | n/a | 4.28 |
| DO | WT STC | No | No | 1.41 | 1.58 | 0.2 | 0.723 | 0.5 | 1.95 | 4.7 | 0.007 | 20.5 |
| DA | WT STC | No | No | 1.41 | 3.2 | 0.3 | 2.22 | 0.3 | 7.98 | 4.54 | n/a | 15.9 |
| DO | WT STC | Yes | Yes | 1.31 | 2.00 | 0.2 | 1.78 | 0.4 | 2.16 | 4.51 | 0.009 | 20.9 |
| DA | WT STC | Yes | Yes | 1.58 | 3.36 | 0.2 | 1.57 | 0.2 | 23.5 | 4.33 | n/a | 4.55 |
| DO | Δ domain III TCC | Yes | Yes | 1.28 | 2.12 | 0.4 | 2.25 | 0.6 | 1.81 | 4.49 | 0.01 | 21.2 |
| DA | Δ domain III TCC | Yes | Yes | 1.31 | 2.85 | 0.2 | 2.32 | 0.3 | 5.14 | 4.33 | n/a | 19.5 |
| DO | Δ domain III STC | Yes | Yes | 1.36 | 2.51 | 0.4 | 2.38 | 0.5 | 2.46 | 4.53 | 0.01 | 20.7 |
| DA | Δ domain III STC | Yes | Yes | 1.63 | 3.25 | 0.1 | 1.75 | 0.1 | 27.0 | 4.33 | n/a | 2.67 |
| DO | WT STC, 60C | Yes | Yes | 1.46 | 1.91 | 0.3 | 2.95 | 0.8 | 3.59 | 4.42 | 0.01 | 16.5 |
| DA | WT STC, 60C | Yes | Yes | 2.13 | 2.99 | 0.1 | 2.20 | 0.1 | 14.2 | 4.26 | n/a | 7.76 |
| DO | Δ domain III STC, 60C | Yes | Yes | 1.51 | 1.73 | 0.1 | 2.13 | 0.3 | 4.10 | 4.38 | 0.009 | 17.4 |
| DA | Δ domain III STC, 60C | Yes | Yes | 1.78 | 2.71 | 0.07 | 2.57 | 0.09 | 16.7 | 4.26 | n/a | 7.63 |

**Figure 6 – figure supplement 1**

Fluorescence lifetime fits across all samples. Each sample was fit as the sum of one gaussian and one discrete lifetime. A donor only fluorophore configuration indicates that the Atto647N FRET acceptor fluorophore was omitted from the target DNA. In the case of DNA only and Donor only samples, no variables were held constant. In samples with transpososomes present, the discrete lifetime from the matching DNA-only sample was held constant (and thus has no associated error) in order to account for an unbound/unbent/singly-labeled fraction. FWHM: Full width at half maximum. TCC: target capture complex, CDCs lacking the 3’OH strand transfer nucleophile were used. STC: strand transfer complex, CDCs fully capable of strand transfer were used. α refers to the pre-exponential factor for the decay component. DO, Donor fluorophore only; DA, Donor and Acceptor fluorophores present.
